# Relationship between Heart Rate Variability and Functional Fitness in Breast Cancer Survivors: A Cross-Sectional Study

**DOI:** 10.3390/healthcare9091205

**Published:** 2021-09-13

**Authors:** Alexandre D. Martins, João Paulo Brito, Rafael Oliveira, Tiago Costa, Fátima Ramalho, Rita Santos-Rocha, Nuno Pimenta

**Affiliations:** 1 Institute of Santarém, Sports Science School of Rio Maior–Polytechnic, 2140-413 Rio Maior, Portugal; jbrito@esdrm.ipsantarem.pt (J.P.B.); rafaeloliveira@esdrm.ipsantarem.pt (R.O.); tiagocdacosta@gmail.com (T.C.); fatimaramalho@esdrm.ipsantarem.pt (F.R.); ritasantosrocha@esdrm.ipsantarem.pt (R.S.-R.); npimenta@esdrm.ipsantarem.pt (N.P.); 2 CIEQV—Life Quality Research Centre, Av. Dr. Mário Soares No. 110, 2040-413 Rio Maior, Portugal; 3 CIDESD—Research Centre in Sport Sciences, Health Sciences and Human Development, 5001-801 Vila Real, Portugal; 4 CIPER—Interdisciplinary Centre for the Study of Human Performance, Faculty of Human Kinetics (FMH), University of Lisbon, Estr. da Costa, Cruz Quebrada, Oeiras, 1495-751 Lisboa, Portugal

**Keywords:** breast cancer, heart rate variability, fitness, exercise, parasympathetic nervous system

## Abstract

Background: Breast cancer is the most common malignancy among women worldwide. The treatments may also cause neuromuscular and skeletal disorders; therefore, the aim of this study was to verify the existence of a relationship between heart rate variability and different functional fitness parameters in women survivors of breast cancer. Methods: This cross-sectional study included 25 women survivors of breast cancer, with a mean ± SD age, height, and body mass of 50.8 ± 8.8 years, 1.6 ± 0.7 m, and 67.1 ± 12.3 kg, respectively. Patients underwent measurements of heart rate variability with time and frequency domain analyses, as well as a “30 s chair-stand test”, “6 min walking test”, “timed up and go test”, and “ball throwing test”. Results: A multiple linear regression analysis showed that from the heart rate variability frequency domain, high frequency explained 21% (R^2^ = 0.21) of the “30 s chair-stand test” performance. Conclusion: The findings of this study highlight high frequency as a predictor of “30 s chair-stand test” performance, regardless of age and time after diagnosis, suggesting its usefulness as a clinical indicator of functionality in breast cancer survivors. This study presents a straightforward and non-invasive methodology predicting functional fitness in women breast cancer survivors potentially applicable to clinical practice.

## 1. Introduction

Oncological diseases such as breast cancer (BC) are a global public health problem with high incidence and mortality in developed countries. The high incidence and mortality make BC one of the main public health problems worldwide [1]. The BC mortality rate has been decreasing due to advances in treatments, although the survival of a cancer patient varies greatly according to the type of cancer, the stage, and the age at diagnosis [2]. In Portugal, data from 2020 revealed that 7.041 cases of BC were diagnosed, with 1.864 patients unable to survive [3].

Despite the advances and the importance of cancer treatment for patient survival, there are often side effects caused by such treatments, including dysfunction of the autonomic nervous system (ANS), which can affect functional fitness and quality of life of cancer survivors [4]. Disfunction of the ANS has been reported to affect about 80% of cancer patients [5], being associated with reduced survival [6]. Various agents used in chemotherapy are known to be associated with cardiovascular events, a phenomenon known as cardiovascular toxicity [7], and seem to have an impact on cardiac autonomic control (e.g., increase in heart rate, a decrease in the time between each heartbeat, and cardiac arrhythmias) [8]. Radiotherapy and surgery often induce more localized anatomical syndromes; that is, some manifestations of the ANS can be induced by damage to a gland or epithelial tissue in the affected surgery or radiotherapy field [4]. The failure of baroreflexes has been pointed out as a consequence of radiation induced in areas such as the neck and chest, due to damage to the carotid sinus and glossopharyngeal nerves [9].

The ANS takes on special importance in the autonomic control of the heart at rest, in orthostatic situations, in thermal regulation, or during the practice of physical exercise [10], through both the sympathetic nervous system (SNS) and the parasympathetic nervous system (PNS). Heart rate variability (HRV) can indicate the PNS activity [11,12] and has the potential to provide additional information on a variety of clinical conditions [11,13].

HRV is a promising non-invasive clinical marker used to assess the regulation of cardiac function based on the variations in the temporal distance between two peaks of the R waves (RR interval) of the QRS complex as assessed by an electrocardiogram [14]. Since the establishment of the *European Society of Cardiology and the North American Society of Pacing and Electrophysiology* guidelines [11], HRV has acquired a special relevance in the study of ANS [14,15] and is considered a prognostic tool with clinical relevance [15] because it can provide an index of the PNS [11]. Low HRV indicates reduced capacity for adaptation, while high HRV suggest a good capacity for adaptation and efficient autonomic mechanisms [13]. Low HRV at rest is suggested to reflect low activation of the PNS as compared to the SNS in the regulation of cardiac function and has been reported to trigger harmful physiologic responses such as increased inflammation, promoting increased oxidative stress [16] and metastatic dissemination [17] in cancer patients. Furthermore, Arab et al. (2016) [6] found evidence of chemotherapy and surgery having important effects on the imbalance between the SNS and the PNS. The improvement of vagus nerve activity, which is the main nerve of the PNS, may allow enhanced cancer treatment finetuning, identifying patients at risk of secondary complications; therefore, it has been identified as an important area of research [4,18].

Cancer treatments may also cause neuromuscular and skeletal disorders [19]. Accordingly, several studies have reported loss of functional fitness in breast cancer survivors (BCS) after treatments [20,21]. Functional fitness impairments lead to physical inactivity [22], which causes loss of muscle strength and a state of constant fatigue [23], affecting the social activities of these patients [24], which together have the capacity to cause further impairments.

In the oncological setting, it has become clear that exercise is a safe and effective supportive therapy in the management of several treatment-related side effects and in improving overall physical fitness [25,26]. The impacts of some supportive therapy modalities such as physical exercise on vegetative function seem to improve HRV. This is due to potential mechanisms associated with decreased activation of the sympathetic nervous system and increased activation of the parasympathetic nervous system [27,28,29]. Some studies reported that moderate aerobic endurance activity improves autonomic cardiac regulation in cancer patients both during and after acute cancer treatment. This regulation, therefore, might be considered as an additional benefit of the overall individualized cancer therapy [28,29,30]. Some authors [31,32,33] have analyzed the efficacy of aerobic exercise and observed positive effects on systemic outcomes (cardiac output, resting HR, and bodyweight), reductions in peak VO2, and increases in troponin levels; however, several factors may modify the interpretation of HRV responses to exercise [34]. It is commonly observed that HRV responses are affected by relative exercise intensity. Specifically, HRV measures are often observed to decrease during and after exercise at a moderate intensity [35,36,37]. The first ventilation threshold has been suggested to demarcate an autonomic ‘binary threshold’ [38].

Overall, in a recent position paper, the European Society of Cardiology task force for cancer treatments and cardiovascular toxicity suggested the possible utility of aerobic exercise as a promising strategy to attenuate the cardiotoxicity of treatments [39].

The present study arose from the unmet need to create a straightforward, replicable, and non-invasive approach, which for this population is essential and would relate functional fitness and cardiac autonomic control; therefore, the aim of the present study was to verify the existence of a relationship between vagus nerve activity assessed by HRV and different functional fitness parameters in women survivors of BC after adjusting for potential confounders.

## 2. Materials and Methods

### 2.1. Participants

Based on a priori sample size calculation for linear multiple regression (α level = 0.05 and number of predictors = 3) through G-Power software [40], 20 participants are required to achieve a sample size power of 95.2%; thus, the present study included 25 volunteer female BC survivors (50.80 ± 8.80 years) followed in a hospital in Lisbon, Portugal. The oncologist was provided with the eligibility assessment and gave clearance to participate in the study. Then, all women provided a face-to-face interview to confirm the data. Each volunteer was informed about the study’s aims and potential benefits and risks. Finally, all participants gave their written informed consent to be enrolled in the study.

The study was approved by the ethics committee of the Polytechnic Institute of Santarém (number: 042020) and was carried out in accordance with the World Medical Association’s Declaration of Helsinki for human studies.

The following inclusion criteria were applied: (i) women aged between 30 and 69 years; (ii) primary diagnosis of BC confirmed by biopsy (stage I to IIIa); (iii) no history of surgical or non-surgical treatment before the cancer diagnosis; (iv) not pregnant. The exclusion criteria were as follows: (i) presence of active metastases; (ii) presence of tumors; (iii) presence of cardiovascular problems (e.g., heart failure before tumor diagnosis); (iv) presence of other diseases (e.g., diabetes, hypertension, depression) [41].

Subjects in the sample were considered inactive if they did not meet at least one of the 3 criteria of the IPAQ-SF, defined as achieving the minimum recommended to be considered minimally active [42].

### 2.2. Procedures

The same procedures from our previous study [41] were applied, namely all participants answered a sociodemographic questionnaire (anamnesis) regarding the type of tumor, treatments performed, year of tumor diagnosis, current medication, type of surgery, and tumor stage. All assessments were performed in the morning, in a clinical facility with an ambient temperature and relative humidity of 22–23 °C and 50–60%, respectively, by the same researchers in order to minimize possible measurement errors. All pre-test instructions given to ensure subjects safety and comfort before administering the health-related fitness test were provided before arrival at the testing facility.

#### 2.2.1. Anthropometric and Body Composition Assessment

The first assessment included the measurement of weight and height through a scale and stadiometer (SECA 220, Hamburg, Germany) to the nearest 0.01 kg and 0.1 cm, respectively. To perform this assessment, participants were asked to use light clothes, without shoes, and to be standing in the Frankfort horizontal position. Body mass index (BMI) values were calculated using the standard formula (BMI = body mass (kg)/height^2^ (m^2^)). The World Health Organization classification was used to classify participants into “underweight”, “normal weight”, “overweight”, and “obese” [43].

Then, the BIA assessment was applied using a multifrequency tetrapolar InBody^®^ S10 instrument (Model JMW140, Biospace Co., Ltd., Seoul, Korea) following the manufacturer’s instructions and comprehensive guidelines described elsewhere [41] to obtain PhA values in degrees (°) at 50 kilohertz. This assessment was performed with participants in the supine position with their arms and legs abducted at a 45° angle, while the surfaces of the right hand and foot dorsal were cleaned with alcohol. After a 10 min rest in a room without noise, eight electrodes were placed on the cleaned surfaces and measurements were performed. For descriptive purposes, we considered a value of <5.6° to diagnose increased risk of survival [44].

For both anthropometric and body compositions assessments, participants were asked to not ingest caffeine or alcohol during the 12 h prior to the assessments and they were only assessed if they were in the luteal phase of ovulatory menstrual cycles. Otherwise, they waited until being in the luteal phase.

#### 2.2.2. Heart Rate Variability Assessment

We followed directives from the *Task Force of the European Society of Cardiology and the North American Society of Pacing and Electrophysiology* [11] and recommendations from Laborde et al. (2017) [15]. For the measurement of HRV, the participants remained in the dorsal decubitus position, in a noise-free space and with an ambient temperature of 22–23 °C for 10 min. The first 5 min of collection were excluded (stabilisation period) and the remaining 5 min were used to calculate the variables in the time and frequency domains.

The HR was recorded beat-by-beat during the experimental protocol via a HR monitor (Polar^®^ RS800Cx, Kempele, Finland) [45] with a sampling rate of 1 kilohertz (kHz). The inter-beat intervals recorded were then transferred to the Polar Precision Performance Software (v.3.0, Polar^®^ Electro, Finland), which permits visualization of HR and signal stability. Intervals were recorded and saved as “.txt” files. Next, digital filtering was achieved using the same Polar Precision Performance Software supplemented with manual filtering for the elimination of artifacts. To facilitate calculation of the HRV indices, we enforced the Kubios HRV software package (Kubios HRV, Biomedical Signal Analysis Group, Department of Applied Physics, University of Kuopio, Finland) [46].

In the time domain, the following indices were calculated: (i) mean RR (mean of the RR intervals in ms); (ii) SDNN (standard deviation of RR intervals in ms); (iii) RMSSD (root mean square of successive RR interval differences in ms); (iv) pNN50 (percentage of successive RR intervals that differ by more than 50 ms). For the frequency domain, the following indices were calculated: (i) LF (absolute power of the low-frequency band, 0.04–0.15 Hz, in ms^2^); (ii) HF (absolute power of the high-frequency band, 0.15–0.4 Hz, in ms^2^; (iii) ratio of LF-to-HF power (LF/HF). The stress index is the square root (to make the index normally distributed) of Baevsky’s stress index, which was previously proposed in [47] and described in detail in the Kubios software manual [48].

#### 2.2.3. Functional Fitness Assessment

Participants performed an initial 10 min warm up of low intensity walking with 5 min of stretching exercises for major muscle groups before the functional fitness assessment. Then, functional fitness was assessed through standardized tests often used in BCS [49], which included a 30 s chair-stand test (starting from the sitting position, the participant stood and sat as fast as possible within 30 s, after which the number of repetitions was recorded) [50]; timed up and go test (TUG test, which started from the sitting position, then the participant stood, performed a 3 m lap around a cone, then returned to the sitting position, after which the time was recorded) [51]; ball throw test (from a sitting position, the participant was asked to throw a 3 kg ball as far as possible in three attempts, in which the best attempt was recorded in meters) [52]; 6 min walking test (6MWT, which consisted of walking as fast as possible without running for 6 min, after which distance in meters was recorded) [53].

### 2.3. Statistical Analysis

Descriptive statistics were used to characterize the sample. The Shapiro–Wilk test was used to test the normality of the results. The results are presented as means ± standard deviation (SD). The relationship between dependent and independent variables after controlling for age and time after diagnosis was verified using semipartial correlations [54] (Pearson product–moment correlation coefficient (*r*)), allowing the magnitudes of the associations to be determined (*r* = 0.10 to 0.29—small; *r* = 0.30 to 0.49—moderate; *r* = 0.50 to 1—strong) [54]. When age or time after diagnosis was the independent variable, the correlation was controlled for tumor size and age or time after diagnosis.

Multiple linear regression was conducted, using the Enter method, between dependent variables (functional tests) correlated with the independent variables (HRV parameters) variables to predict patients’ functional fitness after adjusting for confounding variables (age and time after diagnosis) The level of significance was set at *p* < 0.05 (two-tailed), except in the multiple regression analysis, in which a *p* < 0.1 was accepted to indicate the independent variable was a significant predictor, within the model [55]. All data were analyzed using IBM SPSS Statistics (version 22, IBM Corporation (SPSS Inc., Chicago, IL, USA).

## 3. Results

Data regarding participant characteristics can be found in Table 1. The participants’ BMI values were classified as underweight (n = 0, 0%), normal weight (n = 11, 44%), overweight (n = 8, 32%), or obese (n = 6, 24%). The average BMI of participants was classified as overweight. The phase angle was found to be equal to the 5.6° threshold.

Table 2 summarizes the participants’ clinical data, including the type and stage of cancer and treatment details. Most of the participants were in cancer stage II, did not receive hormonal therapy, and presented with secondary lymphedema.

Table 3 shows the results of the functional fitness tests and of the HRV parameters. The analysis of the relationship between HRV parameters and functional fitness tests showed associations between the HF and “30 s chair-stand test” (r = 0.445; *p* = 0.034 (moderate magnitude)) and between the stress index and “ball throw test” (r = −0.416; *p* = 0.048 (moderate magnitude)). No other HRV parameters were found to be correlated with any functional tests in the present study (Table 4).

Further Pearson correlation tests with HRV parameters showed associations between mean RR and rest heart rate (RHR) (r = −0.523; *p* = 0.011 (strong magnitude)), between RMSSD and RHR (r = −0.447; *p* = 0.032 (moderate magnitude)), and between pNN50 and RHR (r = −0.691; *p* < 0.01 (strong magnitude)). In the frequency domain, an association was observed between the LF and phase angle (r = 0.470; *p* = 0.024 (moderate magnitude)).

Among functional fitness tests, the Pearson correlation values showed associations between the “TUG test” and the “ball throw test” (r = −0.531; *p* = 0.009 (strong magnitude)), “30 s chair-stand test” (r = −0.531; *p* = 0.009 (strong magnitude)), and “6MWT” (r = −0.485; *p* = 0.019 (moderate magnitude)).

Regarding functional fitness, further Pearson correlation tests showed associations between the “TUG test” and time after diagnosis (r = 0.408; *p* = 0.050 (moderate magnitude)); and between the “ball throw test” and weight (r = 0.454; *p* = 0.029 (moderate magnitude)), height (r = 0.416; *p* = 0.049 (moderate magnitude)), and muscle mass (r = 0.620; *p* = 0.002 (strong magnitude)). Finally, an association was shown between “6MWT” and height (r = 0.512; *p* = 0.013 (strong magnitude)).

Multiple linear regression analysis was performed by adjusting the relationships between HF and the “30 s chair-stand test” and between the stress index and “ball throw test” for confounding variables (Table 5). The HF alone explained 21% of the performance in the “30 s chair-stand test” (β = 0.46; *p* = 0.021). On the other hand, the stress index alone could not explain the performance in the “ball throw test”; however, after adjustment for confounding variables, it explained about 36% of the performance (β = −0.37; *p* = 0.048).

## 4. Discussion

To our knowledge, this is the first study to analyze the relationship between vagus nerve activity as assessed by HRV and different functional fitness parameters in BCS. The participants were all women, with an average age of 51 years old, which seems rather predominant in BC [41,44], and had a BMI classified as overweight, which is in line with what has been observed in BCS of the same age [6,41]. The studied participants showed an average PhA of 5.6°, which is considered to be borderline for diagnosing higher risk for lower survival in BC [44]. This highlights the importance of carefully monitoring these BC patients and both finding and using comprehensive clinical markers that may better inform interventions and enhance follow-up of these persons.

Regarding vagus nerve regulation as assessed by HRV, specifically SDNN and RMSSD, in the time domain, as well as LF, HF, and LF/HF ratio in the frequency domain, the results of the present study are in line with a previous study carried out on cancer survivors [56]. The values reported in the present study and in the previous study are considerably low, particularly when compared with proposed values for healthy persons [56]. Overall, the results for HRV parameters reveal lower parasympathetic regulation or an imbalance in its relation with sympathetic regulation of the cardiac function at rest, which has been acknowledged as an adverse cardiac autonomic control associated with lower survival [6]. The HF is suggested to reflect the activity of the PNS and is related to the effect of the respiratory cycle on heart rate, being an indicator of vagal cardiac control [11,13]. In the present study, the mean value observed for HF (127.08 ± 51.04 ms^2^) confirmed a reduced activity of the PNS at rest, again in line with a previous study [56].

To our knowledge, this is the first study that included the assessment of the stress index in BCS, which represents a significant advance in this area and for this population. This parameter is very sensitive to the sympathetic tone increase [47], which for this population has important implications. The present study reported a partial correlation between the stress index and “ball throw test” (Table 4). The “ball throw test” shows the explosive force and the relationship between muscular power production and HRV, which has been previously studied [52]. It can be interpreted that better performance in the “ball throw test” would mean that the subjects present less commonly observed systemic manifestations in BC (e.g., atrophy of fiber types I and II, changes in metabolic enzyme levels that produce decreased strength and resistance, impact functional limitations, and increase myofibrillar protein degradation induced by tumor factors and cytokines involved in cancer biology, which could be associated with a loss of muscle strength in cancer survivors) [57,58]. Despite being very interesting, these results should be confirmed in future studies.

The mechanisms through which autonomic dysfunction mediates the physical function in BCS have been discussed [18] but remain unclear. According to the model proposed by Smith et al. (2014) [59], there is a relationship between the ANS and muscle function that is mediated at least by the control of blood that is transported to skeletal muscles, through the regulation of cardiac output and through the action on peripheral vascular resistance; therefore, this may impact functional fitness. After treatment, there is an increase in sedentary lifestyle and a reduction in the level of physical activity, which leads to a loss of functional fitness, and in some cases the appearance of metabolic syndrome, which aggravates the cardiometabolic risk arising from the treatment phase [7].

Vigo et al. (2015) [18] confirmed the need to investigate the relationship between physical exercise and ANS to define personalized programs of secondary preventions for this population. In this sense, a previous study [60] analyzed lung cancer survivors and found no correlations between SDNN, RMSSD, and the “6MWT”. In the present study, we found no correlations for any HRV parameters with the “6MWT”. A previous study [61] that included both male and female older adults found HRV parameters to be reduced in persons with lower physical activity levels, specifically SDNN (−0.55; *p* < 0.01), RMSSD (−0.83; *p* = 0.008), and HF (−1.18; *p* < 0.01), when compared to high physical activity levels. They also reported strong correlations between these HRV parameters and the functional fitness tests (“TUG test” and the “30 s chair-stand test”) in persons with different physical activity levels. Our study confirmed these results in BCS, although only for the “30 s chair-stand test”. In the present study, multivariate regression analysis showed that HF explained about 21% of the “30 s chair-stand test” performance variation and was found to be a significant predictor, regardless of age and time after diagnosis. The “30 s chair-stand test” mainly assesses lower limb strength, which has been found to be the main determinant of “6MWT” performance [62]. The assessment of vagus nerve activity through HRV may, therefore, constitute a promising surrogate to preliminarily add information to patients’ functional fitness assessment, as it is simple to assess and use, non-invasive, and non-physically demanding for patients who often report increased levels of fatigue after treatment [18].

The results of the functional fitness tests in the present study underscore the already reported need for cancer survivors to perform physical exercise programs after treatment [19,24,41]. Cheville et al. (2008) [19] identified several physical impairments in more than 90% of 163 patients, which affect quality of life and activities of daily living and increase the risk of falls after treatment [19,24]. Functional tests are argued to have great relevance for this population [19,24,41], as they can reflect impaired muscle strength of the lower extremities, walking ability, and poor balance. Recently, Martins et al. (2021) [41] reported low values in the “TUG test”, “ball throw test”, and “30 s chair-stand test” in BCS with worse health and cellular integrity, expressed by PhA. Accordingly, further use of functional tests is recommended in future investigations.

Although it was not the main aim of the present study, we confirmed that correlations between functional tests are also in line with previous studies [19,24], specifically an association between the “ball throw test” and “TUG test” (*r* = −0.531; *p* = 0.009). The “TUG test” has been regarded as a valid tool to assess balance deficits [19], and balance has been argued to be related to the muscle strength of the lower extremities [19,24]. Lower extremity strength has also been found to be the main predictor of the “6MWT” results [62]; therefore, it is not surprising to find that the “6MWT” showed a correlation with the “TUG test” (*r* = −0.485; *p* = 0.019).

Regarding “6MWT”, this study provides a new reference value (796.78 ± 99.18 m) for BCS when compared to a recent systematic review and meta-analysis in this population [63]. The highest value found by this review study was 636 m [64]. Our study had a value range of between 580 and 961.5 m. The great heterogeneity of characteristics between the patients (e.g., years after diagnosis and types of treatment) may explain these results; however, this result shows that it is possible for BCS to have adequate levels of cardiorespiratory fitness.

Cancer treatments, especially chemotherapy, can cause peripheral neuropathy, and this treatment was widely used by the sample under study. Previous studies have reported that peripheral neuropathy and reduced muscle strength may affect balance in BCS [19,24]. The results and relations between functional tests exposed the side effects of treatments, namely chemotherapy and radiotherapy, specifically in terms of strength and balance. This may be attributed to accentuated decreased gait speed and mobility, reflecting generalized impairment of muscle function and balance [19,24]. The “TUG test” also has a correlation with the “30 s chair-stand test” (*r* = −0.482; *p* = 0.015), suggesting a deficiency in gait, which may increase the risk of falls [19], underlining the association of balance with the muscle strength of the lower extremities [19,24]. The results and relations found in the functional tests highlight the importance of finding simple tools that may be used to estimate the functional status of patients, particularly when the settings and the volitional or fatigue levels of patients are barriers to a thorough functional assessment.

The present study has strengths and limitations. Despite the fact that HRV is considered to be a marker of ANS [11], it is an indirect surrogate and does not measure neuroelectric activity of the ANS. Furthermore, this study focused on a specific clinical sub-population, which imposed particular recruitment challenges. Accordingly, although the sample size calculation showed higher power, the results of this study should be carefully interpreted with special attention paid to HRV variables because finger sensors are not the gold standard tools for accessing ANS [15], although the heart rate monitor used in this study has been validated [45]. Additionally, and according to Table 2, it was possible to show different type of treatments, which may have influenced the results.

Another limitation was related to BMI, which revealed an overweight classification. This means that overweight was a potential confounding factor in this study, reinforcing the need for more studies taking into account this variable.

Even so, the present results represent a relevant preliminary analysis to establish the importance of HRV for the prediction of functional fitness in BCS. Future investigations should strive to increase sample size and should include a follow-up of these variables to assess HRV capacity to predict variations in functional fitness and to assess the responses to interventions involving such variables.

## 5. Conclusions

The findings of this study highlight HF as a predictor of “30 s chair-stand test” performance, regardless of age and time after diagnosis, suggesting its usefulness as a clinical indicator of functionality in BCS; however, since this is the first study that sought to verify this relationship, further studies should be carried out in BCS and in other types of cancer. Finally, the results of functional tests reveal reduced muscle agility and strength, confirming the need for this clinical population to actively participate in physical exercise programs after treatment.

## Figures and Tables

**Table 1:** Demographic and physiologic characteristics of the study participants.

Variables	Mean ± SD
Age (years)	50.80 ± 8.80
Weight (kg)	67.10 ± 12.30
Height (m)	1.60 ± 0.70
BMI (kg/m^2^)	26.30 ± 4.80
Muscle Mass (kg)	23.50 ± 3.76
Phase Angle (°)	5.60 ± 0.42
Body fat (kg)	24.25 ± 9.55
SpO_2_ (%)	97.16 ± 1.41
RHR (bpm)	69.84 ± 8.93
SBP (mmHg)	117.96 ± 14.22
DBP (mmHg)	80.96 ± 6.83
Time after diagnosis (years)	6.84 ± 5.64
Tumor size (mm)	24.68 ± 14.78

Abbreviations: kg, kilograms; m, meters; °, degrees; mmHg, millimeter of mercury; BMI, body mass index; SpO_2_, oxygen saturation level; RHR, rest heart rate; bpm, beats per minute; SBP, systolic blood pressure; DBP, diastolic blood pressure; mm, millimeters.

**Table 2:** Clinical characteristics of study participants organized by treatment type.

Variable	Chemotherapy N (%)	Radiotherapy N (%)	Hormonal Therapy N (%)	Radiotherapy + Chemotherapy N (%)	Radiotherapy + Chemotherapy + Hormone Therapy N (%)
Treatment type	5 (20)	2 (8)	1 (4)	6 (24)	11 (44)
Surgery type					
Mastectomy	5 (20)	1 (4)	1 (4)	5 (20)	10 (40)
Quadrantectomy	0 (0)	1 (4)	0 (0)	1 (0)	1 (4)
Hormonal Therapy					
Aromatase Inhibitors	0 (0)	0 (0)	0 (0)	0 (0)	4 (16)
Tamoxifen	0 (0)	0 (0)	1 (4)	0 (0)	6 (24)
Aromatase + Tamoxifen Inhibitors	0 (0)	0 (0)	0 (0)	0 (0)	1 (4)
Did not perform	5 (20)	2 (8)	0 (0)	6 (24)	0 (0)
Tumor Stage					
I	0 (0)	0 (0)	0 (0)	0 (0)	2 (8)
II	4 (16)	1 (4)	0 (0)	2 (8)	6 (24)
III	1 (4)	0 (0)	1 (4)	2 (8)	3 (12)
IV	0 (0)	1 (1)	0 (0)	2 (8)	0 (0)
Type of Breast Carcinoma					
Ductal carcinoma in situ	2 (8)	0 (0)	1 (8)	2 (8)	2 (8)
Non-Special Invasive Type	3 (12)	2 (8)	0 (0)	2 (8)	4 (16)
Invasive Lobular Carcinoma	0 (0)	0 (0)	0 (0)	2 (8)	4 (16)
Invasive Papillary Carcinoma	0 (0)	0 (0)	0 (0)	0 (0)	1 (4)
Secondary Lymphedema					
Yes	2 (8)	1 (4)	0 (0)	3 (12)	2 (8)
No	3 (12)	1 (4)	1 (4)	3 (12)	9 (36)

**Table 3:** Results of functional fitness tests and HRV parameters.

Variables	Mean ± SD
Functional fitness tests	
30 s chair-stand test (rep)	15.24 ± 3.94
Timed Up and Go test (TUG) (s)	5.87 ± 0.76
Ball Throw Test (m)	1.89 ± 0.62
6 min walking test (m)	796.78 ± 99.18
HRV—Time Domain	
Mean RR (ms)	778.20 ± 157.96
SDNN (ms)	35.78 ± 17.19
RMSSD (ms)	41.31 ± 21.10
pNN50 (%)	10.90 ± 13.62
HRV—Frequency Domain	
LF (ms^2^)	169.12 ± 52.04
HF (ms^2^)	127.08 ± 51.04
LF/HF ratio	1.49 ± 0.56
Stress Index	15.78 ± 17.77

Abbreviations: HRV, heart rate variability; ms, milliseconds; rep, repetitions; s, seconds; m, meters; SDNN, standard deviation of RR intervals; RMSSD, root mean square of successive RR interval differences; pNN50, percentage of successive RR intervals that differ by more than 50 ms; LF, low-frequency band; HF, high-frequency band.

**Table 4:** Pearson correlations between heart rate variability and functional fitness.

Variables	30 s Chair-Stand Test (rep)	Timed Up and Go Test (s)	Ball Throw Test (m)	6 min Walking Test (m)
Mean RR (ms)	0.217	−0.331	−0.238	−0.033
SDNN (ms)	0.267	−0.171	0.183	−0.026
RMSSD (ms)	0.394	−0.165	−0.054	−0.087
pNN50 (%)	0.287	−0.338	0.061	0.040
LF (ms^2^)	0.115	−0.061	0.132	0.056
HF (ms^2^)	0.445 *	−0.004	0.192	−0.035
LF/HF ratio	−0.292	0.072	−0.149	0.081
Stress Index	0.334	0.124	−0.416 *	−0.155

Abbreviations: ms, milliseconds; rep, repetitions; s, seconds; m, meters; SDNN, standard deviation of RR intervals; RMSSD, root mean square of successive RR interval differences; pNN50, percentage of successive RR intervals that differ by more than 50 ms; LF, low-frequency band; HF, high-frequency band; *, *p* < 0.05.

**Table 5:** Multiple linear regressions analysis between dependent and related independent variables.

Model	Unstandardized Coefficients		Standardized Coefficients	Change Statistics	*p*
	**Β**	**Std. Error**	**Β**	**R^2^ Change**	
Model 1 ^a^—SEE = 3.57	-	-	-	0.21	0.021 **
(Constant)	10.74	1.95	-	-	<0.01 ***
HF, ms^2^	0.03	0.01	0.46	-	0.021 **
Model 2 ^a^—SEE = 3.73	-	-	-	0.22	NS
(Constant)	11.68	6.67	0.45		0.094 *
HF, ms^2^	0.04	0.02	−0.03		0.034 **
Age, years	−0.12	0.13	−0.05		NS
Time after diagnosis, years	−0.04	0.20			NS
Model 3 ^b^—SEE = 0.60	-	-	-	0.09	NS
(Constant)	2.05	0.16	-	-	<0.01 ***
Stress Index	−0.10	0.01	−0.30	-	NS
Model 4 ^b^—SEE = 5.53	-	-	-	0.36	0.023 **
(Constant)	3.23	0.82	-	-	0.001 **
Stress Index	−0.01	0.01	−0.37	-	0.048 **
Age, years	−0.02	0.02	−0.25	-	NS
Time after diagnosis, years	−0.04	0.03	−0.32	-	NS

Abbreviations: HF, hight frequency band; ms, milliseconds; SEE, standard error of the estimate; ^a^, 30 s chair-stand test with dependent variable in the following model; ^b^, ball throw test with dependent variable in the following model; NS, not significant *p* > 0.1; *, *p* < 0.1; **, *p* < 0.05; ***, *p* < 0.001.

## Data Availability

The datasets used and analyzed during the current study are available from the corresponding author on reasonable request.
